# The Predictive Value of Baseline Target Lesion SYNTAX Score for No-Reflow during Urgent Percutaneous Coronary Intervention in Acute Myocardial Infarction

**DOI:** 10.1155/2021/9987265

**Published:** 2021-08-04

**Authors:** Guofeng Gao, Han Xu, Dong Zhang, Chenxi Song, Changdong Guan, Bo Xu, Dong Yin, Kefei Dou

**Affiliations:** Department of Cardiology, Cardiovascular Institute, Fuwai Hospital and National Center for Cardiovascular Diseases, Chinese Academy of Medical Sciences & Peking Union Medical College, Beijing 100037, China

## Abstract

**Objectives:**

To evaluate the predictive value of target lesion SYNTAX score (TL-SS) for no-reflow in the patients with acute myocardial infarction undergoing urgent percutaneous coronary intervention (PCI).

**Background:**

Risk assessment, prevention, and prompt management of no-reflow in urgent PCI are crucial but remain challenging. SYNTAX score emerged as a tool for prediction, but may contain redundant information.

**Methods:**

After screening of consecutive patients who underwent urgent PCI in Fuwai Hospital from January 2013 to December 2013, 487 patients with 528 lesions were involved. The endpoint was no-reflow during the PCI procedure.

**Results:**

No-reflow occurred in 52 patients (10.7%) and 53 lesions (10.0%). High TL-SS levels were strongly associated with increased risks of no-reflow in the urgent PCI procedure (all adjusted *P* < 0.05). TL-SS displayed good discrimination ability for no-reflow (C-statistics = 0.76, 95% CI 0.72–0.80), which was better than that of SYNTAX score (*P*=0.016). Following categorizing the lesions into two groups according to the Youden Index, the high-risk group (TL-SS ≥8) showed significantly higher no-reflow rate compared with the low-risk group (TL-SS <8) (20.6% vs. 3.6%, odds ratio 6.86, 95% confidence interval 3.50–13.41, *P* < 0.001). In the target lesions that underwent balloon predilation, maximum predilation pressure >10 atm was associated with higher rate of no-reflow in the high-risk group (odds ratio 3.81, 95% confidence interval 1.10–13.17).

**Conclusions:**

TL-SS is a potential predictor for risk stratification of no-reflow in urgent PCI. In the high TL-SS lesions that underwent balloon predilation, maximum predilation pressure >10 atm was associated with higher risk of no-reflow.

## 1. Introduction

Urgent percutaneous coronary intervention (PCI) is the key treatment for patients with ST-segment elevation myocardial infarction (STEMI) and very-high-risk or high-risk non-ST-segment elevation myocardial infarction (NSTEMI) [1, 2]. However, in a substantial proportion of patients, thrombolysis in myocardial infarction (TIMI) flow grade 3 is not achieved after the PCI procedure, mainly because of the no-reflow phenomenon [3–6]. The no-reflow phenomenon is defined as inadequate myocardial perfusion in the presence of a patent epicardial coronary artery and is caused by microvascular obstruction [6–8]. The no-reflow phenomenon during urgent PCI has been reported to be an independent predictor of adverse events, including increased risk of mortality [4, 9, 10]. Therefore, prediction, prevention, and prompt management of no-reflow are crucial for the urgent PCI procedure. The mechanisms responsible for no-reflow include preexisting coronary microvascular dysfunction, ischemic and reperfusion injury of the ischemic myocardium, distal embolization of the target vessel, and individual susceptibility [5, 8]. Also, the characteristics of the target lesion or the target vessel play a key role for the occurrence of no-reflow [11, 12]. Several studies have demonstrated that SYNTAX score emerged as a tool for prediction of no-reflow in urgent PCI in acute myocardial infarction (AMI) [13–15]. However, SYNTAX score represents the entire complexity of the coronary arteries and is composed of the scores of each lesion, including the lesion which is not infarct related or not intended to undergo intervention. The aim of the present study is to evaluate the predictive value of target lesion SYNTAX score (TL-SS) for no-reflow in the patients with AMI undergoing urgent PCI.

## 2. Materials and Methods

### 2.1. Study Population and Procedure

We screened consecutive patients who were admitted to the hospital with a diagnosis of STEMI or NSTEMI and for whom urgent PCI was performed in Fuwai Hospital from January 2013 to December 2013. Acute myocardial infarction (AMI) is defined based on the Fourth universal definition of myocardial infarction (2018) [16]. STEMI is defined by chest pain suggestive for myocardial ischemia for at least 30 minutes before hospital admission, time from the onset of symptoms of less than 24 hours, and an ECG with new ST-segment elevation in two or more contiguous leads of ≥0.2 mV in leads *V*2–*V*3 and/or ≥0.1 mV in other leads or a probable new-onset left bundle branch block. NSTEMI is defined as in accordance with AMI definition but not in accordance with STEMI definition. Urgent PCI included primary PCI in patients with STEMI, immediate PCI (<2 hours) in patients with very-high-risk NSTEMI, and early PCI (<24 hours) in patients with high-risk NSTEMI in accordance with current guidelines [1, 2]. Exclusion criteria were (a) patients with a history of coronary artery bypass grafting and (b) patients with angiographic evidence of coronary mechanical obstruction after PCI, including residual stenosis >50%, residual dissection, and tissue prolapse. Finally, the present study included 487 patients with 528 lesions.

Dual antiplatelet therapy including aspirin and P2Y12 inhibitors was prescribed before the PCI procedure. In patients who were not taking long-term aspirin, aspirin was administered at a dose of 300 mg before the procedure, and in patients who were not taking long-term P2Y12 inhibitors, clopidogrel 300 to 600 mg or ticagrelor 180 mg loading dose was administrated before the procedure. Unfractionated heparin (100 IU/kg) was administered to all patients during the procedure. The PCI strategy and stent selection were at the discretion of the interventional cardiologists. Following the procedure, dual antiplatelet therapy was recommended for at least 12 months.

This study was approved by the institutional review boards of Fuwai Hospital. Written informed consent was obtained from all patients before the PCI procedure [17].

### 2.2. Data Collection

**Figure 1:**
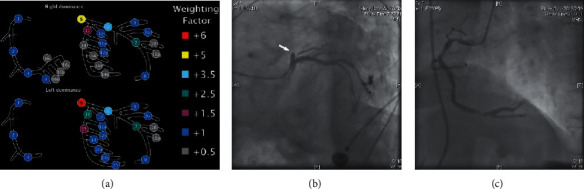
Calculation of the TL-SS. Coronary tree segments and their vessel segment weighting factor based on the presence of a right of left dominant system (a). Also, a multiplication factor of ×2 segment weighting is used for nonocclusive (50–99% diameter stenosis) lesions and ×5 for total occlusive (100% diameter stenosis) lesions. An example of the calculation of the TL-SS in patients with extensive anterior infarction is shown (b and c). The proximal LAD lesion (white arrow), which was total occlusive, was the culprit lesion and target lesion. The occluded proximal LAD led to the segment weighting 3.5 × 5 points (segment 6). The nonvisible mid and distal LAD segments added 2 additional points. The angiographic feature of the lesion did not fulfill the definition of thrombus of SYNTAX score algorithm. Therefore, the final TL-SS was 3.5 × 5 + 2 = 19.5. The lesions within RCA (c) were not the target lesion and scored as nTL-SS. LAD, left anterior descending coronary artery; nTL-SS, nontarget lesion SYNTAX score; RCA, right coronary artery; and TL-SS, target lesion SYNTAX score.

Clinical data were obtained by reviewing the electronic medical records. Blood sampling and echocardiography were performed before the PCI procedure. All baseline and procedural angiograms were analyzed in an independent core laboratory. Calculation of baseline SYNTAX score was performed visually by experienced technicians who were blinded to the procedure outcome using the SYNTAX score algorithm (http://syntaxscore.com/). If interobserver grading differed, consensus was reached after review. SYNTAX score was derived based on each of the lesions in the entire coronary artery tree. Each vessel segment involved in a lesion with a ≥50% diameter stenosis in vessels ≥1.5 mm in diameter was scored and awarded a multiplication factor related to location and severity (Figure 1(a)).Also, more points were added based on further characteristics of the lesion, which included features of total occlusions, bifurcation or trifurcation, ostial lesion, lesion length > 20 mm, severe tortuosity, heavy calcification, and thrombus. The score of the target lesion undergoing PCI was defined as TL-SS (Figure 1(b)). Nontarget lesion SYNTAX score (nTL-SS) was defined as nTL-SS = SS − TL-SS. Angiographic coronary blood flow was assessed at baseline and after PCI on the basis of TIMI flow grade [18]. Data of the procedure were recorded, including predilation, thrombus aspiration, stent diameter and length, postdilation, and dilation pressures. The effectiveness of myocardial perfusion was assessed by myocardial blush grade (MBG) [19].

### 2.3. Endpoint and Definition

The endpoint of the present study was no-reflow during the PCI procedure. Each angiogram was analyzed by both TIMI and MBG scoring. The no-reflow phenomenon was defined as TIMI flow grade <3 and/or MBG <2 without angiographic evidence of mechanical vessel obstruction [6].

### 2.4. Statistical Analysis

Continuous variables were described as the mean ± standard deviation (SD) and compared using Student's *t*-test. Categorical variables were expressed as counts and percentages and compared using chi-squared or Fisher's exact test, as appropriate. TL-SS levels were modelled as both continuous variable and tertiles categorical variable. Univariable analyses were performed with the logistic regression method to calculate the odds ratio (OR) and 95% confidence interval (CI). As the patients' characteristics from the same individual were correlated when using lesion or procedure data, multivariable analyses were performed with general estimated equation analysis by adjusting the variables with statistically significant (*P* < 0.1) comparisons and with clinical consideration to calculate OR and 95% CI. Receiver operating characteristic (ROC) curves were used to estimate the discrimination ability of TL-SS, SS, and nTL-SS. Dichotomous cutoff of the TL-SS was determined by the Youden Index with deLong's method. For additional sensitivity analysis, subgroup analysis was conducted with a logistic regression model. All statistical analyses were performed using SPSS Statistics 25.0 (IBM Corp., Armonk, NY, USA) and SAS version 9.4 (SAS Institute, Cary, NC, USA) at a significance level of two-sided 0.05.

## 3. Results

No-reflow occurred during the PCI procedure of 52 patients (10.7%) and 53 lesions (10.0%). One patient suffered no-reflow twice during 2 urgent PCI procedures in different days. The characteristics of patients, target lesions and procedure, and no-reflow incidence are shown in Supplemental Tables 1 and 2.

The overall patient characteristics and lesion characteristics are shown in Tables 1 and 2, respectively. Patients who suffered no-reflow were more likely to be STEMI, had worse baseline cardiac function, that is, lower ejection fraction, and had higher baseline SYNTAX score. Lesions located in the left circumflex coronary artery were less likely to exhibit no-reflow during the PCI procedure. Also, ostial lesions, lesions with initial TIMI 0–1 flow, and lesions within larger reference diameter vessel were more likely to exhibit no-reflow. No-reflow was associated with balloon predilation, higher maximum predilation pressure, less stent implantation, larger maximum stent diameter, and longer PCI procedural duration.

Univariable analyses showed that higher TL-SS levels were significantly associated with increased risks of no-reflow during the PCI procedure (unadjusted OR 1.14, 95% CI 1.09–1.19, *P*=0.001) (Table 3). Two multivariable analyses models were performed to validate the predictive ability of TL-SS. Model 1 included TL-SS, age, EF, STEMI, LCX location, and reference vessel diameter. In consideration of correlations, the variables included in the algorithm of SYNTAX score calculation were not involved in the multivariable analysis models. Model 2 included 2 more procedural variables, balloon predilation, and thrombus aspiration. Both multivariable analyses models showed that higher TL-SS levels were significantly associated with increased risks of no-reflow(model 1: adjusted OR 1.08, 95% CI 1.03–1.14, *P*=0.001; model 2: adjusted OR 1.08, 95% CI 1.03–1.14, *P*=0.001) (Table 3). The independent predictors of model 2 are displayed in Supplemental Table 3.

**Figure 2:**
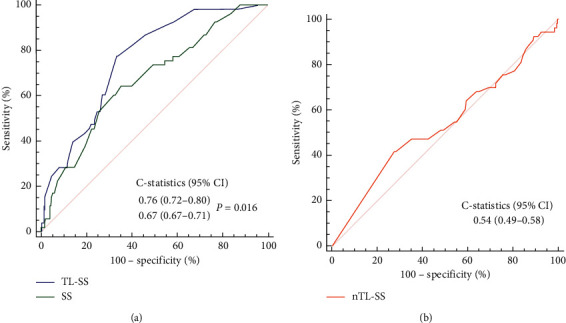
ROC curve analyses estimating the predictive value of TL-SS and SS (a) and nTL-SS (b). nTL-SS, nontarget lesion SYNTAX score; ROC, receiver operating characteristic; SS, SYNTAX score; and TL-SS, target lesion SYNTAX score.

**Figure 3:**
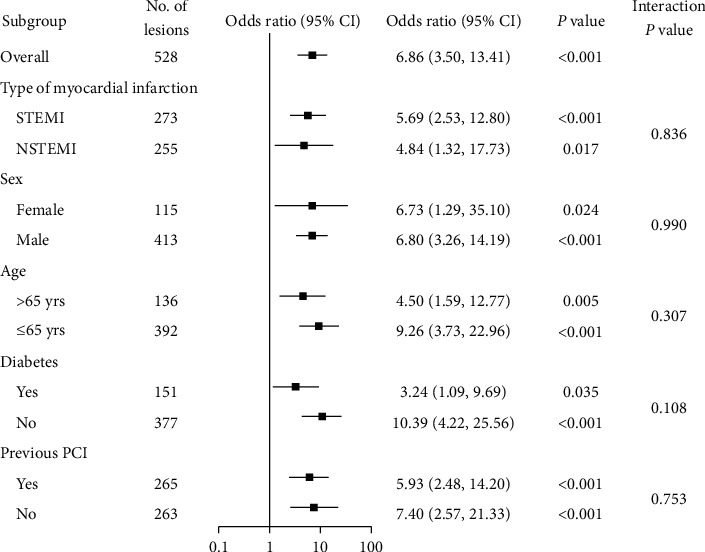
Subgroup analyses are shown with odds ratios and 95% confidence intervals for no-reflow. There were no significant interactions in any of the subgroups (interaction *P* value > 0.1 for all comparisons). CI, confidence interval; NSTEMI, non-ST-segment elevation myocardial infarction; PCI, percutaneous coronary intervention; STEMI, ST-segment elevation myocardial infarction; and TL-SS, target lesion SYNTAX score.

Based on ROC curves analysis, TL-SS displayed good discrimination ability for no-reflow during the PCI procedure (C-statistics = 0.76, 95% CI 0.72–0.80) (Figure 2(a)). Also, the discrimination ability of TL-SS was significantly better than that of SS (C-statistics = 0.76 vs. 0.67 for TL-SS and SS, respectively, *P*=0.016) (Figure 2(b)). By contrast, nTL-SS displayed no discrimination ability for no-reflow based on ROC curves analysis (C-statistics = 0.54, 95% CI 0.49–0.58, *P*=0.382). The best cutoff value of TL-SS according to the Youden Index was 7.5. Then, the entire lesion cohort of this study was categorized into two groups: the low-risk group (TL-SS<8, *n* = 329) and high-risk group (TL-SS ≥8, *n* = 199).The high-risk group showed significantly higher no-reflow rate compared with the low-risk group (20.6% vs. 3.6%, OR 6.86, 95% CI of OR 3.50–13.41, *P* < 0.001). Subgroup analyses showed the result was consistent across subgroups (*P* value < 0.05 in all subgroups) and no significant interactions in any of the subgroups (interaction *P* value > 0.05 for all comparisons) (Figure 3).

**Figure 4:**
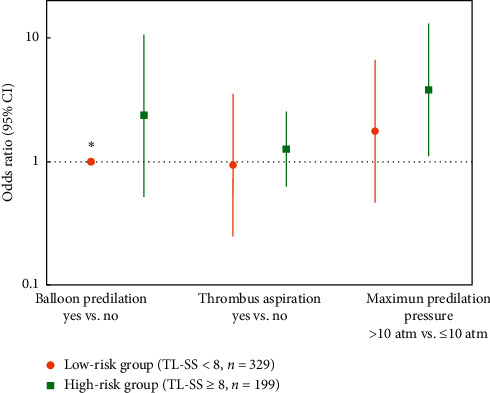
Associations between parameters of lesion preparation and no-reflow occurrence in both high- and low-risk groups. The error bars represent 95% confidence intervals. CI, confidence interval; TL-SS, target lesion SYNTAX score. ^*∗*^Data were not suitable for the logistic regression method and were compared using Fisher's exact test (no-reflow incidence of balloon predilation Yes vs. No, 4.2% vs. 0.0%, *P*=0.386).

Furthermore, we analyzed the association between parameters of lesion preparation and no-reflow occurrence in both high- and low-risk groups. No association was observed between the performance of balloon predilation or thrombus aspiration and the rate of no-reflow in both groups. In the lesions that underwent balloon predilation, maximum predilation pressure >10 atm was associated with higher rate of no-reflow in the high-risk group (OR 3.81, 95% CI 1.10–13.17, *P*=0.034), while no such association was observed in the low-risk group (OR 1.75, 95% CI 0.46–6.63, *P*=0.407) (Figure 4). Maximum predilation balloon diameter and maximum predilation time were not associated with no-reflow in both groups.

## 4. Discussion

The main findings of the present study can be summarized as follows: (1) in this consecutive cohort of AMI patients, TL-SS was one of independent predictors for the no-reflow phenomenon in the urgent PCI procedure; (2) TL-SS demonstrated good discrimination ability, which was significantly better than that of SS; and (3) in the target lesions that underwent balloon predilation, maximum predilation pressure >10 atm was associated with higher rate of no-reflow in the TL-SS ≥8 group.

As the significant association with adverse prognosis of the no-reflow phenomenon, risk estimate of no-reflow is of paramount importance in clinical practice [4, 9, 10]. Risk estimate and stratification before intervention can assist interventional cardiologists for prevention of no-reflow and more prompt management when no-reflow occurs [7]. The characteristics of the target lesion or the target vessel are significantly associated with the occurrence of no-reflow [11, 12]. Also, previous studies adopting intracoronary imaging indicated that the feature of plaque, such as attenuated plaque, thin-cap fibroatheroma, and necrotic core, strongly correlated with no-reflow [20–22]. However, considering the extended procedural duration and increased cost, the extensive application of intracoronary imaging is limited in the urgent PCI procedure. The assessment based on an angiogram can be carried out instantly following angiography and assist interventional cardiologists for strategy making before intervention. As the constant proliferation and iterations of new scoring systems make it impractical for adopting them into clinical practice adequately, the present study aimed to validate the well-established, long-term tool, i.e., the SYNTAX score system [23]. Several studies have demonstrated that SYNTAX score emerged as a tool for prediction of no-reflow in urgent PCI in acute myocardial infarction [13–15]. SYNTAX score represents the entire complexity of the coronary arteries and is composed of the scores of each lesion, including the nontarget lesion which can be represented as nTL-SS. Based on the results of the present study, the predictive ability of TL-SS for no-reflow was significantly better than that of SS. Also, the noneffective discrimination ability of nTL-SS indicated that the nTL-SS might be the redundant information composed in SS for prediction of no-reflow.

SYNTAX score was developed as a tool to systematically analyze the coronary angiogram and to specify the number of coronary lesions, their angiographic location, and anatomical complexity and has been widely applied and validated in the last decade [24, 25]. SYNTAX score is the sum of the scores of all lesions. The present study focused on the score of the lesion requiring treatment, i.e., TL-SS. TL-SS combines the anatomical location, the stenotic severity, and adverse lesion characteristics of the target lesion [24]. The present study demonstrated that TL-SS contained significant predictive information for the no-reflow phenomenon in the urgent PCI procedure, which is consistent with the major mechanisms underlying this phenomenon. The mechanisms that have been shown to contribute to the development of no-reflow include ischemic and reperfusion injury of the ischemic myocardium and distal embolization of the target vessel [5, 8]. Ischemic and reperfusion injury is associated with the time and extent of myocardial ischemia [8]. TL-SS contains the location of the target lesion and its importance in supplying blood to the myocardium, i.e., vessel-segment weighting based on the Leaman score, which can represent the area of the myocardium at risk [26]. The vessel-segment weighting is multiplied by 5 when the lesion is total occlusion or by 2 with 50%–99% stenotic severity. The first segment visible distally of occlusion can represent collateral flow (+1 pre-non-visible segment). The area at risk, baseline TIMI flow, and collateral flow are parallel with the extent of ischemic and reperfusion injury [8]. Also, previous studies have demonstrated the area at risk and baseline TIMI flow to be independent predictive factors of the no-reflow phenomenon in urgent PCI [9, 12, 27]. However, the duration of myocardial ischemia and other factors, such as ischemic preconditioning which plays a cardioprotective role, can affect the ischemic and reperfusion injury and are not included within the angiographic characteristics nor TL-SS [28]. Beside the area at risk and the blood flow, another major mechanism underlying no-reflow in urgent PCI is distal coronary embolization of plaque components and thrombus. Also, mechanical obstruction of microvascular may be accompanied by the inflammatory vascular response and vasospasm [29]. A previous study has demonstrated that angiographic morphologic features of “high-burden thrombus formation” were independent predictors of the no-reflow phenomenon [12]. The occlusion status of the target vessel, blunt stump, thrombus presentation, and lesion length > 20 mm in SYNTAX algorithm may also indicate “high-burden thrombus formation.” Consistent with a previous study, reference target vessel diameter was an independent predictor of no-reflow, but is not involved in SYNTAX score [12]. Because in most cases, the target lesion of AMI is composed of plaque and thrombus, reduced baseline TIMI flow, severity of stenosis, lesion length, and reference target vessel diameter are also associated with plaque burden of the target lesion. Also, as previous studies adopting intracoronary imaging indicated, the feature of plaque, which is difficult to be estimated by angiography, strongly correlated with no-reflow [20–22].

Based on the results of the present study, the incidence rate of no-reflow in STEMI was significantly higher than in NSTEMI, and STEMI was an independent predictor of no-flow after multivariable analyses. The pathogenesis and lesion characteristics of NSTEMI are associated with its lower incidence rate of no-reflow. Compared with STEMI, the pathogenesis of NSTEMI is more heterogeneous, including plaque rupture or erosion, spasm, severe narrowing, and coronary artery dissection [2]. Also, compared with STEMI, culprit lesions in NSTEMI have less plaque rupture and thrombus and small plaque mass [30]. The present study demonstrated that the patients with NSTEMI had higher baseline ejection fraction and the target lesions of NSTEMI displayed better initial TIMI flow, were located with smaller reference diameter vessel, and scored much lower TL-SS (Supplemental Tables 1 and 2). The present study aimed to focus on the generality of the occurrence of no-reflow in the urgent PCI procedure, and the subgroup analyses indicated that TL-SS ≥8 was able to stratify risks both in STEMI and NSTEMI subgroups.

As mentioned above, TL-SS contains the importance of target vessel in supplying blood to the myocardium. Higher TL-SS is associated with higher risk of no-flow; furthermore, when no-reflow occurs, higher TL-SS may be associated with larger area of microvascular obstruction, which contains prognostic value [4, 31]. Further study combining TL-SS and cardiac magnetic resonance is needed to validate this hypothesis. On the other hand, TL-SS contains redundant information for prediction of the no-reflow phenomenon, such severe calcification, bifurcation lesion (Table 2). However, the proportion of lesions with these characteristics and the weight of scores are relatively low.

Prediction of patients at risk for no-reflow before PCI may be beneficial from the perspective of prevention and prompt management [7]. Because the present study was an observational study in real world, the strategy choosing and equipment selection were at the discretion of the interventional cardiologists. The interventional cardiologists would decide whether to implant a stent or perform postdilation and pressure of inflation, based on the coronary flow status in each step of the PCI procedure. Therefore, because of the aforementioned confounders, we investigated the association of different strategies of lesion preparation, which was performed just after angiography, with no-reflow occurrence in high- and low-TL-SS-risk groups. Previous studies indicated that routine deferred stent implantation did not reduce the occurrence of microvascular obstruction and adverse events; however, in high-no-reflow-risk patients, deferred stenting reduced no-reflow and increased myocardial salvage [32–34]. Routine thrombus aspiration did not improve clinical outcomes; however, in high-thrombus-burden patients, the trend was toward reduced cardiovascular death [35]. Also, the use of distal embolic protection decreased the incidence of no-reflow in patients with high risk of distal embolization [36]. These findings suggest that the risk-stratification-guided interventional strategy displays potential benefit for reducing no-reflow and improving clinical outcomes. In the present study, maximum predilation pressure >10 atm was associated with higher rate of no-reflow in the high-risk (TL-SS ≥8) group, but not in the low-risk group. Maximum predilation balloon diameter was not associated with no-reflow. Predilation is performed by many cardiologists with a semicompliant balloon, whose diameters always increase with increasing pressure, particularly at balloon ends. Asymmetrical dilation of the target lesion may facilitate the dislodgment, fragmentation, and distal embolization of the clot or plaque component [37]. Thus, when cardiologists have decided to perform predilation, relative low inflation pressure may be benefit from the decreased incidence of no-reflow.

Limitations: There are several limitations of the present study that should to be noted. First, the present study was a retrospective analysis of an all-comer cohort study in real world. Although a multivariable analysis was performed for significant confounders, it may suffer from residual confounders. Second, the data were derived from a single center, which may introduce selection bias and limit the reliability and generalizability. Third, the predictive ability of TL-SS should be further validated in a different study dataset.

## 5. Conclusions

In conclusion, TL-SS was a strong risk factor for the no-reflow phenomenon in the urgent PCI procedure. Also, TL-SS demonstrated good discrimination ability, which was significantly better than that of SS. In the high-risk target lesions that underwent balloon predilation, maximum predilation pressure >10 atm was associated with higher risk of no-reflow.

## Figures and Tables

**Table 1:** Baseline patient characteristics.

	All (*n* = 487)	Reflow (*n* = 435)	No-reflow (*n* = 52)	*P* value
Age, yrs	58.14 ± 11.68	57.80 ± 11.43	60.96 ± 13.36	0.065
Male	384 (78.9%)	340 (78.2%)	44 (84.6%)	0.281
Body mass index, kg/m^2^	26.13 ± 3.20	26.08 ± 3.29	26.60 ± 2.35	0.155
Diabetes	135 (27.7%)	120 (27.6%)	15 (28.8%)	0.848
Hypertension	299 (61.4%)	265 (60.9%)	34 (65.4%)	0.532
Hyperlipidemia	293 (60.2%)	263 (60.5%)	30 (57.7%)	0.700
Smoking history	324 (66.5%)	289 (66.4%)	35 (67.3%)	0.900
Previous MI	64 (13.1%)	56 (12.9%)	8 (15.4%)	0.612
Previous PCI	100 (20.5%)	92 (21.1%)	8 (15.4%)	0.331
Previous stroke	61 (12.5%)	56 (12.9%)	5 (9.6%)	0.502
Peripheral vascular disease	29 (6.0%)	25 (5.7%)	4 (7.7%)	0.536
COPD	10 (2.1%)	10 (2.3%)	0 (0.0%)	0.610
Creatinine clearance (ml/min)	93.05 ± 26.55	93.45 ± 27.06	89.74 ± 21.85	0.342
Ejection fraction (%)	56.11 ± 7.63	56.75 ± 7.31	50.74 ± 8.11	<0.001
EF < 40% or HF	32 (6.6%)	22 (5.1%)	10 (19.2%)	0.001
STEMI	263 (54.0%)	221 (50.8%)	42 (80.8%)	<0.001
Hemoglobin (g/L)	141.66 ± 21.07	141.02 ± 20.98	148.78 ± 21.06	0.067
White blood cell count (10^9^/L)	8.93 ± 3.12	8.86 ± 3.04	9.77 ± 3.88	0.146
Platelet count (10^9^/L)	217.66 ± 69.28	218.62 ± 70.66	206.96 ± 51.18	0.403
hs-CRP (mg/L)	7.62 ± 5.27	7.62 ± 5.27	7.57 ± 5.46	0.972
Total cholesterol (mmol/L)	4.43 ± 1.07	4.46 ± 1.07	3.99 ± 0.80	0.153
LDL-C (mmol/L)	2.74 ± 0.90	2.74 ± 0.91	2.59 ± 0.65	0.562
HDL-C (mmol/L)	1.01 ± 0.29	1.01 ± 0.29	1.00 ± 0.21	0.952
Left main disease and/or 3-vessel disease	213 (43.7%)	186 (42.8%)	27 (51.9%)	0.208
Multivessel disease	354 (72.7%)	315 (72.4%)	39 (75.0%)	0.692
Baseline SYNTAX score	14.56 ± 8.04	14.03 ± 7.87	18.98 ± 8.21	<0.001

*Note.* All data are presented as *n* (%) or mean ± SD. COPD, chronic obstructive pulmonary disease; CRP, C-reactive protein; EF, ejection fraction; HDL-C, high-density lipoprotein cholesterol; HF, heart failure; LDL-C, low-density lipoprotein cholesterol; MI, myocardial infarction; PCI, percutaneous coronary intervention; STEMI, ST-segment elevation myocardial infarction.

**Table 2:** Angiographic characteristics of the target lesions and procedure.

	All (*n* = 528)	Reflow (*n* = 475)	No-reflow (*n* = 53)	*P* value
Target vessel location				0.010
LM involved	6 (1.1%)	5 (1.1%)	1 (1.9%)	
LAD^*∗*^	224 (42.4%)	196 (41.3%)	28 (52.8%)	
LCX	96 (18.2%)	94 (19.8%)	2 (3.8%)	
RCA	202 (38.3%)	180 (37.9%)	22 (41.5%)	
Initial TIMI 0∼1 flow	290 (54.9%)	248 (52.2%)	42 (79.2%)	<0.001
Lesion length, mm	25.40 ± 13.81	25.08 ± 13.58	28.25 ± 15.54	0.114
Reference vessel diameter, mm	3.13 ± 0.53	3.10 ± 0.53	3.34 ± 0.53	0.002
Severe calcification	9 (1.7%)	8 (1.7%)	1 (1.9%)	1.000
Bifurcation lesion	67 (12.7%)	57 (12.0%)	10 (18.9%)	0.154
Ostial lesion	11 (2.1%)	7 (1.5%)	4 (7.5%)	0.018
TL-SS	7.99 ± 5.64	7.56 ± 5.42	11.78 ± 6.22	<0.001
Radial approach	482 (91.3%)	432 (90.9%)	50 (94.3%)	0.406
Balloon predilation	463 (87.7%)	412 (86.7%)	51 (96.2%)	0.046
Maximum predilation balloon diameter, mm	2.40 ± 0.32	2.39 ± 0.30	2.41 ± 0.42	0.692
Maximum predilation pressure, atm	13.35 ± 4.17	13.17 ± 4.21	14.78 ± 3.58	0.009
Thrombus aspiration	165 (31.3%)	144 (30.3%)	21 (39.6%)	0.166
Stent implantation	489 (92.6%)	444 (93.5%)	45 (84.9%)	0.024
Number of stents per lesion >1 stent implanted	125 (23.7%)	110 (23.2%)	15 (28.3%)	0.403
Total stent length, mm	29.55 ± 14.26	29.15 ± 14.04	33.49 ± 15.93	0.052
Maximum stent diameter, mm	3.18 ± 0.51	3.15 ± 0.50	3.40 ± 0.52	0.003
Balloon postdilation	313 (59.3%)	288 (60.6%)	25 (47.2%)	0.058
Maximum balloon diameter, mm	3.22 ± 0.69	3.21 ± 0.67	3.33 ± 0.79	0.229
Maximum balloon: reference	1.03 ± 0.15	1.03 ± 0.15	1.00 ± 0.18	0.127
Maximum pressure, atm	16.40 ± 3.70	16.39 ± 3.70	16.55 ± 3.77	0.766
Glycoprotein IIb/IIIa inhibitor therapy	116 (22.0%)	104 (21.9%)	12 (22.6%)	0.901
Contrast volume, ml	159.01 ± 77.28	158.60 ± 77.16	162.24 ± 78.96	0.756
IVUS use	8 (1.5%)	7 (1.5%)	1 (1.9%)	0.574
PCI procedural duration, min	39.46 ± 27.14	38.46 ± 27.17	48.38 ± 25.45	0.012

*Note.* All data are presented as *n* (%) or mean ± SD. IVUS, intravascular ultrasound; LAD, left anterior descending coronary artery; LCX, left circumflex coronary artery; LM, left main coronary artery; PCI, percutaneous coronary intervention; RCA, right coronary artery; TL-SS, target lesion SYNTAX score. ^*∗*^There were 3 target lesions involving proximal LCX without involving LM.

**Table 3:** Univariable and multivariable analysis of the association between TL-SS and no-reflow.

TL-SS level, categorical/continuous	Number of patients with no-reflow (%)	Unadjusted OR (95% CI)	Unadjusted *P* value	Adjusted OR (95% CI) (model 1)	Adjusted *p* value (model 1)	Adjusted OR (95% CI) (model 2)	Adjusted *P* value (model 2)
Low tertile (*n* = 155)	1 (0.6%)	Reference	—	Reference	—	Reference	—
Median tertile (*n* = 207)	20 (9.7%)	16.47 (2.64–147.76)	0.007	8.92 (1.08–73.55)	0.042	8.91 (1.11–71.78)	0.040
High tertile (*n* = 166)	32 (19.3%)	36.78 (4.96–272.77)	<0.001	15.13 (1.92–119.09)	0.010	14.81 (1.91–114.78)	0.010
Per 1 score	—	1.14 (1.09–1.19)	<0.001	1.08 (1.03–1.14)	0.001	1.08 (1.03–1.13)	0.001

*Note.* Covariates of model 1: TL-SS, age, EF, STEMI, LCX location, and reference vessel diameter. Covariates of model 2: TL-SS, age, EF, STEMI, LCX location, reference vessel diameter, balloon predilation, and thrombus aspiration. CI, confidence interval; EF, ejection fraction; LCX, left circumflex coronary artery; OR, odds ratio; PCI, percutaneous coronary intervention; STEMI, ST-segment elevation myocardial infarction; TL-SS, target lesion SYNTAX score.

## Data Availability

The raw processed data required to reproduce these findings cannot be shared at this time as the data also form part of an ongoing study.

## Supplementary Materials

Supplementary Materials Supplemental Tables file contains 3 supplemental tables. Supplemental Table 1: baseline patient characteristics according to STEMI and NSTEMI. Supplemental Table 2: angiographic characteristics of the target lesions and procedure according to STEMI and NSTEMI. Supplemental Table 3: independent predictors of no-reflow.
