# Supplementary material for: The Predictive Value of Baseline Target Lesion SYNTAX Score for No-Reflow during Urgent Percutaneous Coronary Intervention in Acute Myocardial Infarction
Source: J Interv Cardiol. 2021 Aug 4;2021:9987265. doi: 10.1155/2021/9987265 (PMC8357514; doi:10.1155/2021/9987265)
Supplement: Supplementary Materials — Supplemental Tables file contains 3 supplemental tables. Supplemental Table 1: baseline patient characteristics according to STEMI and NSTEMI. Supplemental Table 2: angiographic characteristics of the target lesions and procedure according to STEMI and NSTEMI. Supplemental Table 3: independent predictors of no-reflow. [file 9987265.f1.docx]

Supplemental Table 1 Baseline patients’ characteristics according to STEMI and NSTEMI

|  | STEMI (n=263) | NSTEMI (n=224) | P Value |
| --- | --- | --- | --- |
| Age, yrs | 58.11±12.19 | 58.16±11.07 | 0.965 |
| Male | 211 (80.2%) | 173 (77.2%) | 0.420 |
| Body mass index, kg/m^2^ | 26.31±3.23 | 25.93±3.16 | 0.184 |
| Diabetes | 64 (24.3%) | 71 (31.7%) | 0.070 |
| Hypertension | 153 (58.2%) | 146 (65.2%) | 0.114 |
| Hyperlipidemia | 160 (60.8%) | 133 (59.4%) | 0.743 |
| Smoking history | 180 (68.4%) | 144 (64.3%) | 0.333 |
| Previous MI | 34 (12.9%) | 30 (13.4%) | 0.880 |
| Previous PCI | 44 (16.7%) | 56 (25.0%) | 0.024 |
| Previous stroke | 31 (11.8%) | 30 (13.4%) | 0.594 |
| Peripheral vascular disease | 19 (7.2%) | 10 (4.5%) | 0.200 |
| COPD | 3 (1.1%) | 7 (3.1%) | 0.198 |
| Creatinine clearance, ml/min | 93.59±26.06 | 92.42±27.17 | 0.628 |
| Ejection fraction, %  EF < 40% or HF | 53.98±7.44  21 (8.0%) | 58.61±7.07  11 (4.9%) | <0.001  0.172 |
| Hemoglobin, g/L | 144.86±20.51 | 139.33±21.21 | 0.019 |
| White blood cell count, 10^9^/L | 10.78±3.35 | 7.59±2.09 | <0.001 |
| Platelet count, 10^9^/L | 216.30±80.67 | 218.65±59.87 | 0.762 |
| hs-CRP, mg/L | 4.72±4.15 | 8.44±5.27 | <0.001 |
| Total cholesterol, mmol/L | 4.43±1.38 | 4.43±0.96 | 0.992 |
| LDL-C, mmol/L | 2.85±1.16 | 2.71±0.81 | 0.419 |
| HDL-C, mmol/L | 1.07±0.41 | 0.99±0.24 | 0.182 |
| Left main disease and/or 3-vessel disease | 123 (46.8%) | 90 (40.2%) | 0.144 |
| Multi-vessel disease | 198 (75.3%) | 156 (69.6%) | 0.164 |
| Baseline SYNTAX Score | 16.32±7.89 | 12.48±7.73 | <0.001 |
| No-reflow | 42 (16.0%) | 10 (4.5%) | <0.001 |

Note: All data are presented as n (%) or mean ± SD. Note: All data are presented as n (%) or mean ± SD. COPD, chronic obstructive pulmonary disease; CRP, C-reactive protein; EF, ejection fraction; HDL-C, High-density lipoprotein cholesterol; HF, heart failure; LDL-C, Low-density lipoprotein cholesterol; MI, myocardial infarction; NSTEMI, non-ST-segment elevation myocardial infarction; PCI, percutaneous coronary intervention; STEMI, ST-segment elevation myocardial infarction.

Supplemental Table 2 Angiographic characteristics of target lesions and procedure according to STEMI and NSTEMI

|  | STEMI (n=273) | NSTEMI (n=255) | P Value |
| --- | --- | --- | --- |
| Target-vessel location  LM involved  LAD *  LCX  RCA | 1 (0.4%)  111 (40.7%)  44 (16.1%)  117 (42.9%) | 5 (2.0%)  113 (44.3%)  52 (20.4%)  85 (33.3%) | 0.050 |
| Initial TIMI 0~1 flow | 224 (82.1%) | 66 (25.9%) | <0.001 |
| Lesion length, mm | 25.07±13.12 | 25.76±14.52 | 0.566 |
| Reference vessel diameter, mm | 3.18±0.53 | 3.06±0.53 | 0.011 |
| Severe Calcification | 6 (2.2%) | 3 (1.2%) | 0.506 |
| Bifurcation lesion | 30 (11.0%) | 37 (14.5%) | 0.225 |
| Ostial lesion | 6 (2.2%) | 5 (2.0%) | 0.849 |
| Radial approach | 241 (88.3%) | 241 (94.5%) | 0.011 |
| Balloon pre-dilation | 235 (86.1%) | 228 (89.4%) | 0.244 |
| Maximum pre-dilation balloon diameter, mm | 2.40±0.34 | 2.39±0.29 | 0.716 |
| Maximum pre-dilation pressure, atm | 13.57±3.84 | 13.11±4.48 | 0.242 |
| Thrombus aspiration | 135 (49.5%) | 30 (11.8%) | <0.001 |
| Stent implantation | 249 (91.2%) | 240 (94.1%) | 0.202 |
| Number of stents per lesion  >1 stent implanted | 1.17±0.63 | 1.24±0.62 | 0.221 |
| Total stent length, mm | 28.94±13.61 | 30.17±14.91 | 0.342 |
| Maximum stent diameter, mm | 3.22±0.51 | 3.13±0.50 | 0.050 |
| Balloon post-dilation | 154 (56.4%) | 159 (62.4%) | 0.165 |
| Maximum balloon diameter, mm | 3.25±0.73 | 3.19±0.64 | 0.297 |
| Maximum balloon: reference | 1.02±0.16 | 1.04±0.13 | 0.125 |
| Maximum pressure, atm | 16.38±3.76 | 16.42±3.65 | 0.904 |
| Glycoprotein IIb/IIIa inhibitor therapy | 65 (23.8%) | 51 (20.0%) | 0.291 |
| Contrast volume, ml | 156.09±76.52 | 162.71±78.28 | 0.372 |
| IVUS use | 3 (1.1%) | 5 (2.0%) | 0.492 |
| PCI procedural duration, min | 40.15±25.14 | 38.72±29.16 | 0.545 |
| TL-SS | 9.74±6.02 | 6.27±4.77 | <0.001 |
| No-reflow | 43 (15.8%) | 10 (3.9%) | <0.001 |

Note: All data are presented as n (%) or mean ± SD. IVUS, intravascular ultrasound; LAD, left anterior descending coronary artery; LCX, left circumflex coronary artery; LM, left main coronary artery; NSTEMI, non-ST-segment elevation myocardial infarction; PCI, percutaneous coronary intervention; RCA, right coronary artery; STEMI: ST-segment elevation myocardial infarction; TL-SS, target lesion SYNTAX score.

* There were 3 target-lesions involving proximal LCX without involving LM.

Supplemental Table 3 Independent predictors of no-reflow

|  | OR (95% CI) | P value |
| --- | --- | --- |
| TL-SS * | 1.08 (1.03-1.13) | 0.001 |
| STEMI | 3.33 (1.40-7.90) | 0.006 |
| EF † | 0.95 (0.91-0.99) | 0.016 |
| Reference vessel diameter ‡ | 2.05 (1.07, 3.93) | 0.032 |

Note: Derived from general estimated equation model 2.

Covariates of model 2: TL-SS, age, previous PCI, EF, STEMI, LCX location, reference vessel diameter, balloon pre-dilation, thrombus aspiration.

CI, confidence interval; EF, ejection fraction; LCX, left circumflex coronary artery; OR, odds ratio; PCI, percutaneous coronary intervention; STEMI, ST-segment elevation myocardial infarction; TL-SS, target lesion SYNTAX score.

* Per 1 TL-SS point

† Per 1%

‡ Per 1mm
